# Radiographic markers of breast cancer brain metastases: relation to clinical characteristics and postoperative outcome

**DOI:** 10.1007/s00701-021-05026-4

**Published:** 2021-10-22

**Authors:** Anna Michel, Thiemo Dinger, Marvin Darkwah Oppong, Laurèl Rauschenbach, Cornelius Deuschl, Yahya Ahmadipour, Daniela Pierscianek, Karsten Wrede, Jörg Hense, Christoph Pöttgen, Antonella Iannaccone, Rainer Kimmig, Ulrich Sure, Ramazan Jabbarli

**Affiliations:** 1 Department of Neurosurgery and Spine Surgery, University Hospital Essen, University Duisburg-Essen, Hufelandstraße 55, 45147 Essen, Germany; 2 DKFZ Division Translational Neurooncology at the West German Cancer Center (WTZ), DKTK Partner Site, University Hospital Essen, Essen, Germany; 3 Department of Radiology, University Hospital Essen, Essen, Germany; 4 Department of Medical Oncology, University Hospital Essen, Essen, Germany; 5 Department of Radiotherapy, University Hospital Essen, Essen, Germany; 6 Department of Obstetrics and Gynecology, University Hospital Essen, Essen, Germany

**Keywords:** Breast cancer, Brain metastases, MRI necrosis, HER2

## Abstract

**Objective:**

Occurrence of brain metastases BM is associated with poor prognosis in patients with breast cancer (BC). Magnetic resonance imaging (MRI) is the standard of care in the diagnosis of BM and determines further treatment strategy. The aim of the present study was to evaluate the association between the radiographic markers of BCBM on MRI with other patients’ characteristics and overall survival (OS).

**Methods:**

We included 88 female patients who underwent BCBM surgery in our institution from 2008 to 2019. Data on demographic, clinical, and histopathological characteristics of the patients and postoperative survival were collected from the electronic health records. Radiographic features of BM were assessed upon the preoperative MRI. Univariable and multivariable analyses were performed.

**Results:**

The median OS was 17 months. Of all evaluated radiographic markers of BCBM, only the presence of necrosis was independently associated with OS (14.5 vs 22.5 months, *p* = 0.027). In turn, intra-tumoral necrosis was more often in individuals with shorter time interval between BC and BM diagnosis (< 3 years, *p* = 0.035) and preoperative leukocytosis (*p* = 0.022). Moreover, dural affection of BM was more common in individuals with positive human epidermal growth factor receptor 2 status (*p* = 0.015) and supratentorial BM location (*p* = 0.024).

**Conclusion:**

Intra-tumoral necrosis demonstrated significant association with OS after BM surgery in patients with BC. The radiographic pattern of BM on the preoperative MRI depends on certain tumor and clinical characteristics of patients.

**Supplementary Information:**

The online version contains supplementary material available at 10.1007/s00701-021-05026-4.

## Introduction

The breast cancer [8] is one of the most frequent primary cancer entities in women with high impact of interest and prognostic value [6, 9, 53, 68]. Therapy concepts of BC impacting the patients’ survival include the surgical and (neo-) adjuvant treatment, the conventional chemotherapy, endocrine therapy, and radiation, as well as targeted therapy [18, 25, 34, 43, 46, 55, 56, 69, 72].

Depending on different risk factors and applied treatment, 15–50% of BC patients develop brain metastases [5, 10, 23, 35, 38]. The receptor status (RS) plays an important role for therapy concepts and the prognosis of breast cancer brain metastases (BCBM) patients [40, 49, 51, 53, 56]. Individuals with triple negative BC and positive status of human epidermal growth factor receptor 2 (HER2) are prone to BM [35, 38, 51]. The overall survival (OS) after BM surgery depends on multiple factors like Karnofsky Performance Status (KPS) scale score, number of BM, presence of extracranial metastases, patients’ age, timing between BC and BM, histopathological parameters, and (neo-) adjuvant treatments [3, 11, 20, 31, 33, 35, 60, 61]. In case of BCBM, the median OS varies between 7.2 and 37.7 months. [29, 33, 60]

Magnetic resonance imaging (MRI) is a sensitive diagnostic tool and increases the detection rate of BM [10, 26, 38, 57]. Moreover, MRI is commonly used to plan treatment and to control the cancer disease [2, 22, 36, 39, 50, 52, 65]. Recent studies showed that radiographic markers might have additional clinical value for the prognostication of postoperative survival in patients with lung and breast cancer [1, 7, 8, 12, 13, 15, 24, 45]. As to BCBM, the contrast-enhanced T1-weighted MRI features were identified as prognostic factors for therapeutic response after Gamma Knife radiosurgery [73]. In this context, the patient and tumor characteristics associated with the radiographic pattern of BM on MRI are also of clinical relevance. In particular, leptomeningeal infiltration of BM was more common in individuals with HER2-positive and triple-negative BC. [28, 30, 41]

To address the clinical value of radiographic markers of BCBM, we analyzed the association between various radiographic characteristics of BM on the preoperative MRI with demographic, clinical, and immunohistochemical features of BCBM patients selected for surgery. A special attention was drawn on the potential prognostic value of radiographic markers of BCBM for OS.

## Material and methods

This study was performed in accordance with the Declaration of Helsinki and approved by the local ethics committee of the University Hospital Essen (local registration number: 17–7855-BO).

### Patient population

All female patients (age ≥ 18 years) who underwent BM surgery in our institution from January 2008 to December 2019 were included. The cases with missing preoperative MRI were excluded (*n* = 9). Treatment strategy and allocation to BCBM surgery was discussed in the institutional interdisciplinary tumor conference. Common criteria for BM surgery were the size and the mass effect from the lesion(s), presence of considerable perifocal edema and/or neurological symptoms, non-eloquent location, and the KPS score.

### Data management

For the evaluation of the radiographic parameters of BCBM, the T1-, T2-, and contrast-enhanced-weighted images of the preoperative MRI scans were reviewed by the first author (A.M., blinded at this time to all clinical, histological, and survival data) for the presence of following radiographic characteristics of BM: number (single vs multiple), size (maximal diameter), and location (supratentorial vs infratentorial) of BM; intra-tumoral hemorrhage; contrast enhancement (CE) configuration; cystic components; necrosis; edema; midline shift; dural affection; and the relation to the ventricles.

Then, certain clinical and histological features of BCBM patients were recorded from the electronic health records: age (at BC and BM diagnosis), the type of BC surgery (mastectomy vs breast-preserving surgery (BPS)), trastuzumab therapy of BC, the time interval between the diagnosis of BC and BM, preoperative KPS scale, extracranial metastases, RS of BM and BC (hormone receptors: estrogen (ER), progesterone (PR), and HER2), and the receptor conversion (RC) in BM, as well as OS upon the available follow-up data. Moreover, two laboratory parameters at admission were also included for further correlations as commonly evaluated laboratory markers for disease progression and survival in BC patients: white blood cells [16, 27, 44, 47] and lactate dehydrogenase [37, 49, 67]

### Statistical analysis

Data were analyzed using SPSS (version 27, SPSS Inc., IBM, Chicago, IL, USA) statistical software. The variables were reported in median values and interquartile ranges (IQR) between 25 and 75%, or as number of cases (with percentage), as appropriate. The significance level for the *p* value was set at ≤ 0.05. Continuous data were dichotomized according to the established criteria or using the associations in the receiver operating characteristic (ROC) curves. In particular, WBC > 10 × 10^9^/L was referred as leukocytosis and LDH as pathologically increased. The patients’ age was dichotomized at 65 years. In line with the previous studies [59], the size of peri-tumoral edema in the preoperative MRI scans was dichotomized at 10 mm.

First, the associations between preoperative MRI characteristics and patients’ demographic, clinical, immunohistochemical, and laboratory parameters were evaluated in univariate analysis using the chi-square (*χ*2 test) or Fisher exact tests. Significant associations from the univariable analysis were transferred to multivariable binary logistic regression analysis to control for confounders.

The associations between the radiographic markers and OS were evaluated in the univariable and multivariable Cox regression analysis in the same manner. To visualize the survival differences for major study results, the Kaplan–Meier survival plots and log-rank test were performed.

## Results

### Patient population

**Fig. 1:**
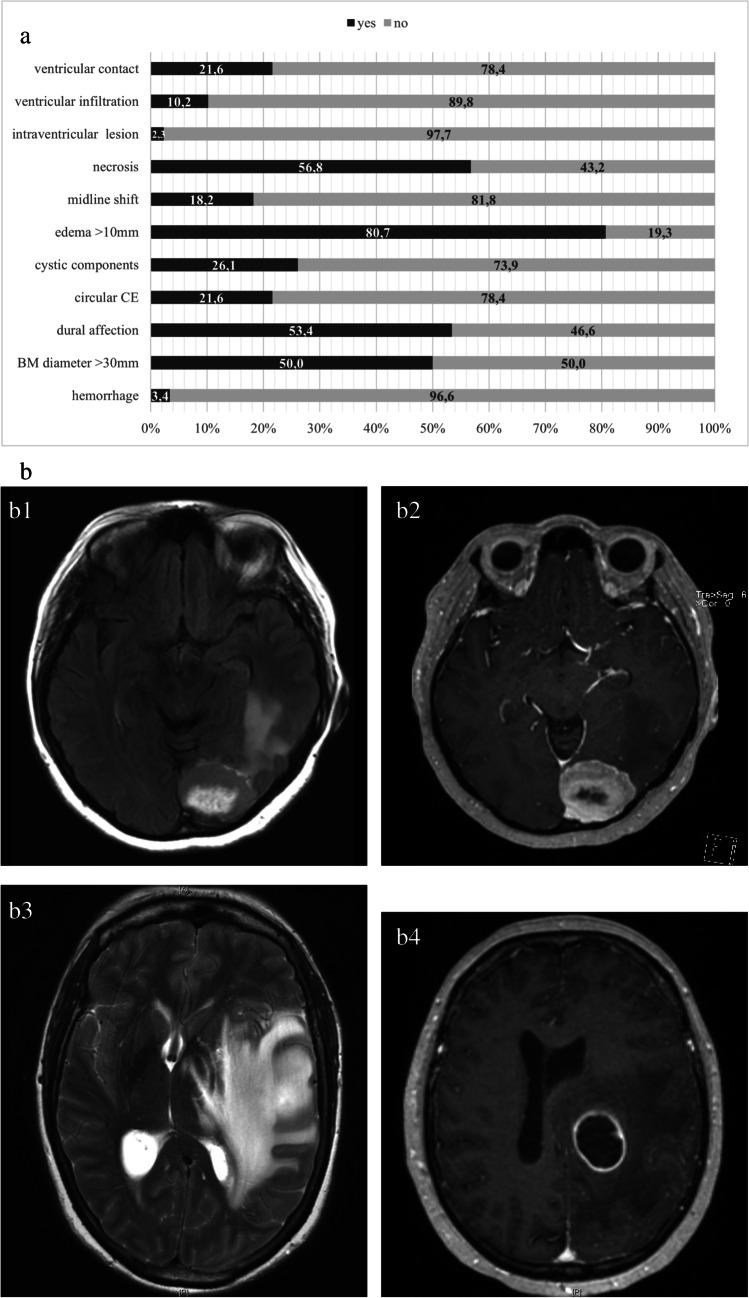
Radiological information’s in preoperative MRI. **a** Preoperative radiological parameters of operated BCBM patients. The following distribution of radiological characteristics are available: ventricular contact (19/88, 21.6%), ventricular infiltration (9/88, 10.2%), intraventricular lesion (2/88, 2.3%), necrosis (50/88, 56.8%), midline shift (16/88, 18.2%), edema > 10 mm (71/88, 80.7%), cystic components (23/88, 26.1%), circular CE (19/88, 21.6%), dural affection (47/88, 53.4%), BM diameter > 30 mm (44/88, 50.0%), and hemorrhage (3/88, 3.4%). **b** Preoperative MRI scans: b1 and b2 demonstrate central necrosis (hash symbol), perifocal edema is seen in b3 (black arrowhead) and b4 shows the circular CE exemplary. Abbreviation: CE, contrast enhancement; BM, brain metastases

The final cohort consisted of 88 female patients. The median OS after BM surgery was 17.0 (7.0–34.8) months. The initial treatment of BC included BPS and trastuzumab in 44 (50%) and 26 (29.5%) cases, respectively. Adjuvant radiotherapy was the standard therapy after BCBM resection. In some cases, system therapy was also adapted. In our cohort, 77 (87.5%) received postoperative radiotherapy. Positive HER2 RS in the BM was identified in 36 cases (40.9%). Table 1 summarizes the major baseline demographic, clinical, and histological characteristics of the patients in the final cohort. On the preoperative MRI scans, 42 patients (47.7%) showed singular and supratentorial BM. The detailed information on the radiographic features of BCBM is presented in the Fig. 1.

**Table 1:** Baseline characteristics of BCBM patients

Parameter	Median (IQR) or Nr. (%)
Clinical parameters	
Number of patients	88 (100%)
OS (months)	17.0 (7.0–34.8)
Preoperative KPS ≥ 80%	77 (87.5%)
Age at BC diagnosis (years)	65.0 (45.0–62.0)
Age at BM diagnosis (years)	55.0 (51.0–68.8)
Time interval BC to BM (months)	42.0 (22.0–100.0)
Number of BM	
*Singular*	58 (65.9%)
*Multiple*	30 (34.1%)
BM location	
*Supratentorial*	55 (62.5%)
*Infratentorial*	33 (37.5%)
Surgical treatment of BC	
* Mastectomy*	42 (47.7%)
* Breast-preserving surgery (BPS)*	46 (52.3%)
Trastuzumab therapy of BC	26 (29.5%)
Adjuvant radiotherapy of BM	77 (87.5%)
Extracranial metastases	35 (39.8%)
Immunohistochemically parameters	
BM HER2 RS	
* Positive*	36 (40.9%)
* Negative*	52 (59.1%)
BM ER RS	
* Positive*	45 (51.1%)
* Negative*	43 (48.9%)
BM PR RS	
* Positive*	17 (19.3%)
* Negative*	71 (80.7%)
HER2 RC	
* Identic*	69 (78.4%)
* Converted*	9 (10.2%)
HR RC	
* Identic*	39 (44.3%)
* Converted*	39 (44.3%)

Abbreviations: *Nr.* number of cases, *BC* breast cancer, *BM* brain metastasis, *IQR* interquartile ranges 25–75%, *OS* overall survival, *RS* receptor status, *HER2* human epidermal growth factor receptor 2, *ER* estrogen receptor, *PR* progesterone receptor, *HR* hormone receptors (= ER and PR), *RC* receptor conversion

### Association between MRI markers and other patients’ characteristics

#### Univariable analysis

Intra-tumoral *hemorrhage* was more frequent in individuals with poor KPS scale (< 80%) at admission (*p* = 0.040).

Moreover, younger age at BC diagnosis (*p* = 0.033), BC therapy with trastuzumab (*p* = 0.019), infratentorial BM (*p* = 0.027), and positive HER2 RS in BM (*p* = 0.017) were associated with *dural affection* in the preoperative MRI.

*Circular CE* was identified more commonly in older patients at BC diagnosis (*p* = 0.001), in patients without trastuzumab therapy (*p* = 0.048), and with negative HER2 RS in BC (*p* = 0.050). Then, negative HER2 (*p* = 0.017) and ER (*p* = 0.001) RS in BM was also associated with circular CE in BCBM.

*Cystic components* in BM were detected more often in BM with negative ER RS (*p* = 0.001).

BM with *necrosis* in MRI showed associations with poorer initial clinical condition (*p* = 0.053), trastuzumab therapy for BC (*p* = 0.046), shorter time interval between BC and BM (*p* = 0.009), negative ER RS in BM (*p* = 0.049), and higher rate of leukocytosis at admission (*p* = 0.007).

BCBM patients without extracranial metastases (*p* = 0.027), shorter time interval between BC and BM manifestation (*p* = 0.024), and negative ER RS in BM (*p* = 0.030) as well as identic HR status in BC and BM (*p* = 0.047) showed more often BM with *perifocal edema* > *10 mm*.

Finally, none of the patients’ characteristics showed significant associations with the *relation of BM to the ventricles* (see supplementary table 1 and 2).

#### Multivariable analysis

For *dural affection*, the following associations remained significant: supratentorial location of (aOR 3.10, 95% CI 1.16–8.27, *p* = 0.024) and positive HER2 RS in BM (aOR 3.30, 95% CI 1.26–8.62, *p* = 0.015). Age ≥ 65 years at BC diagnosis (aOR 5.66, 95% CI 1.18–27.14, *p* = 0.030) and negative ER RS in BM (aOR 21.84, 95% CI 2.37–201.49, *p* = 0.007) were significantly associated with *circular CE*. Moreover, two baseline parameters remained significant in the multivariable analysis for the predictors of *necrosis* in preoperative MRI: time interval between BC and BM (< 3 years, aOR 3.10, 95% CI 1.08–8.85, *p* = 0.035) and preoperative leukocytosis (aOR 3.44, 95% CI 1.19–9.94, *p* = 0.022). Finally, negative ER RS in BM (aOR 3.78, 95% CI 0.99–14.43, *p* = 0.05) was the only parameter independently associated with peri-tumoral *edema* in the multivariable analysis (see Table 2).

**Table 2:** Multivariate analysis of radiological features with clinical, immunohistochemically, and laboratory parameters

Parameter	*p-*value	aOR	95% CI
Dural affection			
Age at BC diagnosis ≥ 65 years	0.097	2.80	0.83–9.41
BM location supratentorial	0.024	3.10	1.16–8.27
BM HER2 RS negative	0.015	3.30	1.26–8.62
Circular CE			
Age at BC diagnosis ≥ 65 years	0.030	5.66	1.18–27.14
Trastuzumab BC therapy	0.425	2.21	0.32–15.48
BC HER2 RS negative	0.969	1.06	0.06–18.75
BM HER2 RS negative	0.651	1.90	0.12–30.84
BM ER RS negative	0.007	21.84	2.37–201.49
Necrosis			
KPS < 90%	0.118	2.35	0.80–6.84
Trastuzumab BC therapy	0.301	1.82	0.58–5.70
TI BC-BM < 3 years	0.035	3.10	1.08–8.85
BM ER RS negative	0.268	1.80	0.64–5.08
Preop. WBC (> 10/nL)	0.022	3.44	1.19–9.94
Edema > 10 mm			
Extracranial metastases	0.108	0.35	0.09–1.26
TI BC-BM < 5 years	0.106	2.83	0.80–10.00
BM ER RS negative	0.052	3.78	0.99–14.43
HR RC identic	0.091	3.19	0.83–12.31

Abbreviations: *BC* breast cancer, *BM* brain metastasis, *RS* receptor status, *HER2* human epidermal growth factor receptor 2, *ER* estrogen receptor, *HR* hormone receptors (= ER and PR (progesterone receptor)), *CE* contrast enhancement, *TI* time interval, *KPS* Karnofsky Performance status, *Preop*. preoperative, *aOR* adjusted odds ratio, *CI* confidence interval

### Association between MRI markers and OS

**Fig. 2:**
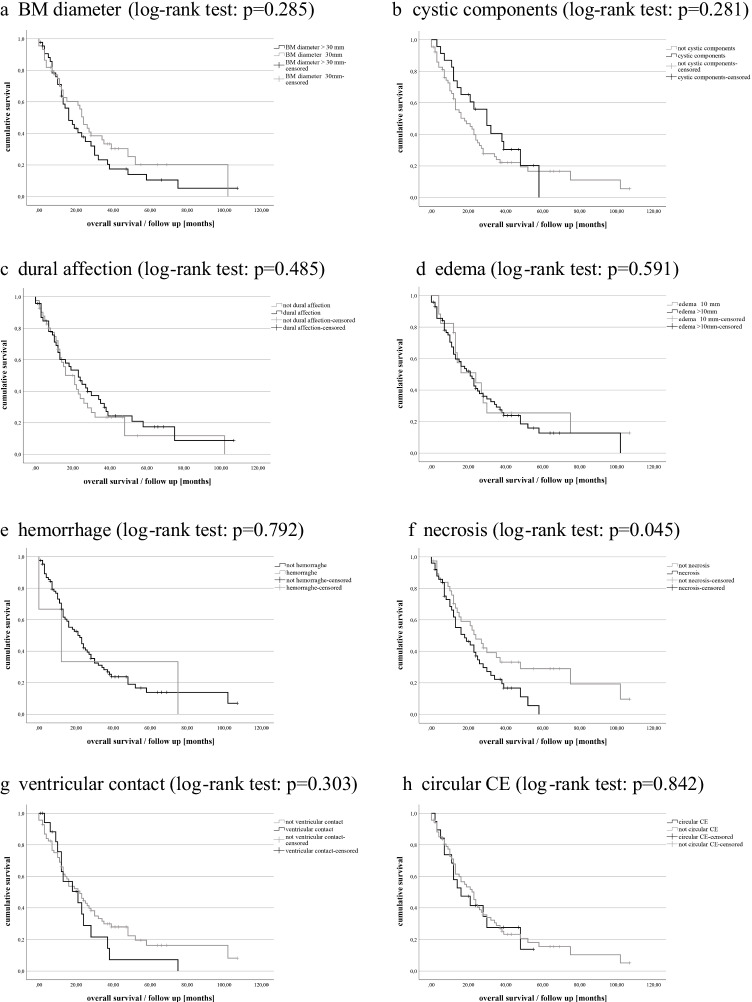
Prediction for OS in patients with operated BCBM: Kaplan Meier curves demonstrate the radiological parameters and their influence on OS. Only necrosis presents as independent prognostic factor for OS for operated BCBM patients (necrosis status in preoperative MRI, log-rank test: *p* = 0.045). **a** BM diameter (log-rank test: *p* = 0.285), **b** cystic components (log-rank test: *p* = 0.281), **c** dural affection (log-rank test: *p* = 0.485), **d** edema (log-rank test: *p* = 0.591), **e** hemorrhage (log-rank test: *p* = 0.792), **f** necrosis (log-rank test: *p* = 0.045), **g** ventricular contact (log-rank test: *p* = 0.303), and **h** circular CE (log-rank test: *p* = 0.842). Abbreviations: BM, brain metastasis; RS, receptor status; HER2, human epidermal growth factor receptor 2; KPS, Karnofsky Performance status; Preop., preoperative

Univariable analysis: Patients with BM *necrosis* showed poorer outcome (median OS 14.50 vs 22.50 months, *p* = 0.051). Other radiographic parameters showed no significant associations with OS (Fig. 2). As to the remaining patient and tumor characteristics, only the positive HER2 RS in BM (median OS 23.5 vs 13.5 months, *p* = 0.017) and favorable preoperative KPS scale (≥ 80%, median OS 22.00 vs 7.00 months, *p* = 0.001) showed significant associations with OS (see supplementary table 3).

**Fig. 3:**
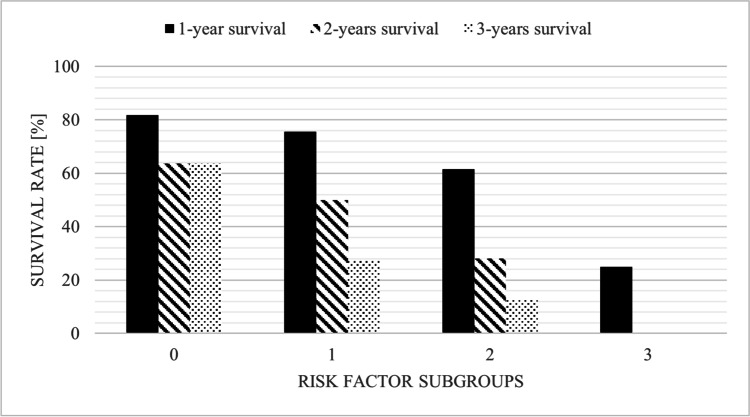
Significant survival predictors in operated BCBM patients. BM HER2 negative RS, preoperative KPS < 80%, and necrosis in preoperative MRI are predictors for poor outcome. Prognostic relevant predictors demonstrate (1 year, 2 years, 3 years) survival rates [in %] in different subgroups (0, 1, 2, 3 risk factors). Abbreviations: BM, brain metastases; HER2, human epidermal growth factor receptor 2; KPS, Karnofsky Performance status; neg, negative; pos, positive

In the final multivariable Cox regression analysis, MRI necrosis (aHR 1.78, 95% CI 1.07–2.96, *p* = 0.027), negative HER2 RS in BM (aHR 1.88, 95% CI 1.10–3.21, *p* = 0.020), and poor preoperative KPS scale scores (aHR 3.33, 95% CI 1.57–7.06, *p* = 0.002) were confirmed as independent predictors for poor OS after BCBM surgery (See Table 3). Figure 3 visualizes the association between the number of present independent predictors and patients’ survival at 1, 2, and 3 years.

**Table 3:** Multivariate analysis for independent predictors of OS after BCBM surgery

Parameter	aHR	95% CI	*p*-value
MRI necrosis	1.78	1.07–2.96	0.027
BM HER2 RS negative	1.88	1.10–3.21	0.020
Preop. KPS < 80%	3.33	1.57–7.06	0.002

Abbreviations: *aHR* adjusted hazard ratio, *CI* confidence interval, *RS* receptor status, *BM* brain metastases, *HER2* human epidermal growth factor receptor 2, *KPS* Karnofsky Performance status, *preop* preoperative, *MRI* magnetic resonance imaging, *OS* overall survival

## Discussion

Currently, MRI is the standard of care in the diagnosis and the evaluation of treatment response in patients with BM. Increasing epidemiologic relevance of BC in the developed countries and considerable survival differences necessitate the identification of simple and reliable prognostic markers for BC patients which might help to predict the disease course at its early stage. In this retrospective study, we evaluated the prognostic value of easily assessable radiological markers of BCBM and found that the necrosis in the preoperative MRI scan is independently associated with postoperative survival in BCBM patients.

It is generally accepted that patients’ age, BC subtype, preoperative KPS scale scores, and the presence of extracranial metastases influence the treatment decisions and survival in individuals with BCBM [3, 11, 31–33, 35, 42, 51, 60]. The location and the number of BM are also relevant parameters for treatment decision and prognosis. So, infratentorial BM were associated with higher morbidity and complications rates in surgical series. [14, 63, 70] Different risk scores based on the patients’ age, KPS scale values, and BC subtype, as well as the patterns of intracranial and extracranial metastases were also confirmed as reliable prognostic markers for BCBM patients [17, 60, 62, 63, 66].

CE MRI is the gold standard in the diagnostics of BM patients and is crucial for the selection of proper treatment strategy. Furthermore, different (MRI-based) imaging characteristics of BM were reported as prognostic markers for survival and treatment response. The radiographic parameters which were previously addressed as clinically relevant markers for cancer patients include the tumor volume; presence of necrotic, perifocal, and cystic components; peri-focal edema; and dural affection, as well as the pattern of CE [4, 8, 15, 54, 58, 59, 64, 71, 73].

Several studies demonstrated the impact of CE-weighted MRIs for the prediction of local tumor control following Gamma Knife radiosurgery and underlined the correlations between EGFR mutation status and clinical aspects with radiological features like CE and mass effect of BM in non-small cell lung carcinoma [13, 15, 19, 21]. However, the data on the clinical value of radiographic BCBM characteristics for the estimation of postoperative survival was still missing.

In the present study, we have identified heterogeneous radiological characteristics of BM which can be easily assessed upon the preoperative MRI imaging without the application of cost- and time-consuming software solutions. We analyzed the relationship between these simple radiographic markers with other baseline parameters and OS of BCBM patients. Of all radiographic BCBM features, only the presence of intra-tumoral necrosis showed independent association with postoperative survival in our cohort. Interestingly, BM necrosis was already reported as prognostic factor for poor local tumor control after Gamma Knife radiosurgery of lung cancer BM [19, 48].

Although the remaining MRI markers of BCBM showed no predictive value for OS, but the observed independent associations with other patient and tumor characteristics might also be of clinical relevance. On one side, BCBM patients with negative ER RS presented more often with circular CE and considerable perifocal edema. On another side, BM with dural affection was more common in HER2-positive and supratentorial BM. In turn, higher rate of tumor recurrence was reported for BM with dural contact [64]. In summary, our findings show that certain histological characteristics (and, possibly, related adjuvant treatment strategies) might influence the radiographic pattern of BCBM.

Accordingly, the observed association between the tumor necrosis and OS in our cohort might be related to certain tumor- and patient-specific characteristics. So, individuals with shorter time interval between BC and BM diagnosis showed more often necrotic components in MRI. Shorter time interval is well established as relevant prognostic factor for BCBM. [20] Another tumor necrosis-related baseline parameter, the leukocytosis at admission, was also previously reported as a significant survival predictor for BC patients [27, 44, 47]. Finally, the RS and preoperative KPS scale scores which showed the associations with the necrosis in univariable analysis are acknowledged survival predictors of BC [31, 40, 51, 60]. For the clarification of the biological background of the association between the tumor features in the MRI scans with the other patients’ characteristics and postoperative survival, further clinical and experimental studies are mandatory.

### Limitations

The retrospective design and the information bias with regard to non-unique technical features of analyzed preoperative MRI scans and partially missing follow-up data are the major limitations of this monocentric study. Moreover, imaging interpretation without the use of threshold-based automated analyses always impairs the risk of investigator bias. Another limitation of our study is the inability of assessment of the extent of metastasis resection with a MRI imaging, since only postoperative computed tomography scans were routinely performed. However, according to the surgical reports, complete resection of BM could be achieved in all cases of the analyzed cohort. Then, the standard perioperative steroid treatment could have impacted the development of leukocytosis. However, the blood sampling and begin of steroid therapy mostly on the admission day lowers the probability of steroid-induced leukocytosis in the analyzed patients. Finally, center-specific selection criteria for BCBM surgery which might vary between the clinics, particularly, in different countries, also limit the generalizability of our results. Therefore, external validation of the analyzed radiographic markers of BCBM is necessary for the clarification of the prognostic value of MRI markers for BCBM patients.

## Conclusion

The radiographic pattern of BCBM on the preoperative MRI depends on certain baseline patient and tumor characteristics like the RS for ER and HER2, patient’s age, time interval between BC and BM diagnosis, and preoperative leukocytosis. In turn, tumor necrosis is independently associated with OS after BCBM surgery. The observed associations between the radiographic tumor characteristics with other clinical and immunohistochemical parameters and patients’ survival might be useful for better understanding of tumor biology in individuals with BCBM.

## Supplementary Information

Below is the link to the electronic supplementary material.

Supplementary file1 (DOCX 37 KB)
