# Supplementary material for: Radiographic markers of breast cancer brain metastases: relation to clinical characteristics and postoperative outcome
Source: Acta Neurochir (Wien). 2021 Oct 22;164(2):439–49. doi: 10.1007/s00701-021-05026-4 (PMC8854251; doi:10.1007/s00701-021-05026-4)
Supplement: Supplementary file 1 — Supplementary file1 (DOCX 37 KB) [file 701_2021_5026_MOESM1_ESM.docx]

**Supplementary table 1:** Associations between radiological parameters with clinical, immunohistochemically and laboratory characteristics

|  | **Hemorrhage BM** | | | **BM diameter >30mm** | | | **Dural affection** | | | **Circular CE** | | |
| --- | --- | --- | --- | --- | --- | --- | --- | --- | --- | --- | --- | --- |
| **Parameter** | **p-value** | **OR** | **95% CI** | **p-value** | **OR** | **95% CI** | **p-value** | **OR** | **95% CI** | **p-value** | **OR** | **95% CI** |
| Age at BC diagnosis ≥65years | 0.094 | 9.33 | 0.79-109.72 | 1.000 | 1.16 | 0.40-3.34 | **0.033** | 0.29 | 0.09-0.91 | **0.001** | 6.86 | 2.14-21.97 |
| Age at BM diagnosis ≥65years | 0.553 | 3.48 | 0.30-40.0 | 1.000 | 0.91 | 0.38-2.15 | 0.276 | 0.60 | 0.25-1.43 | 0.060 | 2.94 | 1.04-8.33 |
| KPS <80% | **0.040** | 16.89 | 1.39-205.32 | 0.196 | 3.04 | 0.75-12.32 | 0.104 | 0.28 | 0.07-1.14 | 1.000 | 0.78 | 0.16-3.98 |
| KPS <90% | 0.065 | 0.92 | 0.83-1.01 | 0.129 | 2.14 | 0.90-5.10 | 1.000 | 0.96 | 0.41-2.25 | 0.602 | 1.40 | 0.50-3.89 |
| Mastectomy as BC therapy | 0.604 | 2.25 | 0.20-25.76 | 0.286 | 1.73 | 0.75-4.03 | 0.834 | 1.11 | 0.48-2.57 | 0.796 | 1.29 | 0.47-3.55 |
| Trastuzumab BC therapy | 1.000 | 1.20 | 0.10-13.84 | 1.000 | 1.00 | 0.40-2.50 | **0.019** | 0.30 | 0.11-0.83 | **0.048** | 0.22 | 0.05-1.04 |
| BC HER2 RS negative | 1.000 | 1.41 | 0.12-16.23 | 0.649 | 1.29 | 0.52-3.18 | 0.067 | 0.40 | 0.16-1.03 | **0.050** | 3.81 | 0.97-14.70 |
| Extracranial metastases | 1.000 | 0.75 | 0.07-8.60 | 1.000 | 1.10 | 0.47-2.58 | 0.081 | 2.32 | 0.96-5.60 | 0.069 | 0.33 | 0.10-1.09 |
| TI BC-BM <1 year | 1.000 | 1.04 | 1.00-1.09 | 1.000 | 1.03 | 0.24-4.39 | 0.141 | 0.27 | 0.05-1.40 | 0.065 | 4.27 | 0.96-19.04 |
| TI BC-BM <3 years | 0.248 | 1.07 | 0.99-1.15 | 0.284 | 1.74 | 0.75-4.06 | 0.669 | 0.78 | 0.36-1.81 | 0.299 | 1.90 | 0.68-5.31 |
| TI BC-BM <5 years | 1.000 | 1.23 | 0.12-14.13 | 0.379 | 1.57 | 0.66-3.77 | 0.515 | 0.74 | 0.31-1.76 | 0.292 | 1.96 | 0.63-6.07 |
| Number of BM >1 | 0.267 | 4.07 | 0.35-46.84 | **0.042** | 0.35 | 0.14-0.89 | 0.499 | 1.50 | 0.61-3.67 | 0.586 | 0.63 | 0.20-1.95 |
| BM location infratentorial | 0.289 | 1.06 | 0.99-1.13 | 1.000 | 1.10 | 0.47-2.61 | **0.027** | 0.34 | 0.14-0.84 | 0.603 | 0.72 | 0.24-2.12 |
| BM HER2 RS negative | 1.000 | 1.40 | 0.12-16.05 | 0.829 | 1.21 | 0.52-2.83 | **0.017** | 0.32 | 0.13-0.79 | **0.017** | 4.89 | 1.31-18.31 |
| BM ER RS negative | 0.112 | 0.93 | 0.86-1.01 | 0.670 | 1.31 | 0.57-3.04 | 0.831 | 0.84 | 0.36-1.94 | **0.001** | 8.30 | 2.21-31.20 |
| BM PR RS negative | 1.000 | 0.96 | 0.91-1.01 | 1.000 | 0.86 | 0.30-2.49 | 0.290 | 1.84 | 0.63-5.40 | 1.000 | 1.36 | 0.35-5.32 |
| HER2 RC converted | 1.000 | 1.14 | 1.05-1.24 | 0.729 | 1.45 | 0.36-5.85 | 0.159 | 3.60 | 0.70-18.59 | 0.676 | 2.22 | 0.26-19.20 |
| HR RC identic | 1.000 | 2.05 | 0.18-23.63 | 0.650 | 0.73 | 0.30-1.79 | 1.000 | 1.12 | 0.46-2.70 | 1.000 | 1.00 | 0.33-3.00 |
| Preop. LDH (>247U/l) | 1.000 | 1.03 | 0.97-1.09 | 0.332 | 0.58 | 0.22-1.53 | 0.808 | 1.20 | 0.46-3.13 | 0.765 | 1.26 | 0.37-4.23 |
| Preop. WBC (>10/nl) | 1.000 | 0.60 | 0.05-6.86 | 1.000 | 1.01 | 0.42-2.41 | 0.824 | 0.84 | 0.35-2.02 | 1.000 | 0.97 | 0.34-2.76 |
|  | **Cystic components** | | | **Necrosis** | | | **Edema >10mm** | | | **Ventricular contact** | | |
| **Parameter** | **p-value** | **OR** | **95% CI** | **p-value** | **OR** | **95% CI** | **p-value** | **OR** | **95% CI** | **p-value** | **OR** | **95% CI** |
| Age at BC diagnosis ≥65years | 0.366 | 1.73 | 0.56-5.39 | 0.789 | 0.82 | 0.29-2.38 | 1.000 | 1.15 | 0.29-4.54 | 1.000 | 0.74 | 0.19-2.89 |
| Age at BM diagnosis ≥65years | 0.617 | 1.41 | 0.53-3.70 | 0.825 | 0.86 | 0.36-2.06 | 1.000 | 1.13 | 0.37-3.39 | 0.296 | 0.52 | 0.17-1.62 |
| KPS <80% | 0.721 | 0.59 | 0.12-2.97 | 1.000 | 0.90 | 0.25-3.20 | 0.684 | 2.62 | 0.31-22.03 | 0.055 | 3.75 | 1.00-14.05 |
| KPS <90% | 0.467 | 1.47 | 0.56-3.83 | **0.053** | 2.46 | 1.00-6.00 | 0.411 | 1.86 | 0.59-5.84 | 1.000 | 1.07 | 0.38-2.98 |
| Mastectomy as BC therapy | 0.363 | 1.27 | 0.49-3.30 | 0.201 | 1.80 | 0.77-4.24 | 0.788 | 0.77 | 0.27-2.23 | 0.068 | 2.99 | 1.02-8.79 |
| Trastuzumab BC therapy | 0.430 | 0.58 | 0.19-1.78 | **0.046** | 2.71 | 1.00-7.37 | 0.768 | 1.46 | 0.43-4.98 | 0.571 | 1.54 | 0.53-4.48 |
| BC HER2 RS | 0.604 | 1.41 | 0.49-4.04 | 0.157 | 0.47 | 0.18-1.22 | 0.410 | 0.59 | 0.18-1.90 | 0.784 | 0.12 | 0.39-3.73 |
| Extracranial metastases | 1.000 | 0.96 | 0.36-2.55 | 0.387 | 1.51 | 0.63-3.62 | **0.027** | 0.28 | 0.09-0.85 | 0.443 | 0.64 | 0.22-1.87 |
| TI BC-BM <1 year | 0.200 | 3.16 | 0.72-13.86 | 0.458 | 2.39 | 0.45-12.56 | 1.000 | 1.78 | 0.20-15.51 | 1.000 | 0.52 | 0.06-4.53 |
| TI BC-BM <3 years | 0.812 | 1.14 | 0.44-2.95 | **0.009** | 3.39 | 1.38-8.32 | 0.058 | 3.34 | 0.99-11.25 | 0.799 | 0.84 | 0.90-2.35 |
| TI BC-BM <5 years | 1.000 | 0.93 | 0.35-2.48 | 0.503 | 1.48 | 0.62-3.55 | **0.024** | 4.00 | 1.31-12.20 | 0.787 | 1.29 | 0.43-3.84 |
| Number of BM >1 | 0.613 | 1.35 | 0.50-3.61 | 0.496 | 1.50 | 0.61-3.72 | 0.780 | 1.30 | 0.41-4.13 | 0.790 | 1.17 | 0.41-3.36 |
| BM location infratentorial | 1.000 | 1.10 | 0.41-2.92 | 0.076 | 2.39 | 0.96-5.93 | 1.000 | 1.13 | 0.37-3.39 | 0.114 | 0.37 | 10.11-1.22 |
| BM HER2 RS negative | 0.325 | 1.84 | 0.67-5.08 | 0.520 | 0.74 | 0.31-1.76 | 0.411 | 0.54 | 0.17-1.69 | 1.000 | 0.94 | 0.34-2.63 |
| BM ER RS negative | **0.001** | 5.76 | 1.90-17.47 | **0.049** | 2.37 | 1.00-5.63 | **0.030** | 3.96 | 1.18-13.34 | 0.607 | 0.71 | 0.25-1.97 |
| BM PR RS negative | 0.218 | 3.15 | 0.66-15.01 | 0.789 | 1.22 | 0.42-3.52 | 0.733 | 1.37 | 0.89-4.90 | 1.000 | 0.87 | 0.45-3.06 |
| HER2 RC converted | 1.000 | 0.81 | 0.15-4.26 | 0.469 | 2.39 | 0.46-12.36 | 0.469 | 2.39 | 0.46-12.36 | 1.000 | 1.12 | 0.21-6.01 |
| HR RC identic | 1.000 | 1.00 | 0.36-2.76 | 0.485 | 1.55 | 0.62-3.87 | **0.047** | 3.89 | 1.13-13.41 | 0.780 | 0.73 | 0.24-2.20 |
| Preop. LDH (>247U/l) | 1.000 | 0.88 | 0.28-2.77 | 0.060 | 2.59 | 0.95-7.05 | 1.000 | 1.14 | 0.36-3.61 | 0.576 | 0.65 | 0.21-1.97 |
| Preop. WBC (>10/nl) | 1.000 | 1.02 | 0.38-2.72 | **0.007** | 3.89 | 1.50-10.09 | 0.057 | 3.35 | 0.90-11.37 | 0.443 | 0.64 | 0.22-1.84 |

Abbreviations: BM: brain metastases, KPS: Karnofsky Performance Status scale, BC: breast cancer, CE: contrast-enhancement, HER2: human epidermal growth factor receptor 2, TI: time interval, ER: estrogen receptor, PR: progesterone receptor, HR: hormone receptors (=ER and PR), RS: receptor status, RC: receptor conversion, OR: odds ratio, CI: confidence interval, preop.: preoperative, WBC: white blood cells, LDH: lactat dehydrogenase

**Supplementary table 2:** Distribution of the analyzed radiographic patterns according to different patient and tumor characteristics.

| **Parameter** | **Hemorrhage BM** | **No hemorrhage BM** | **Parameter** | **Necrosis** | **No necrosis** |
| --- | --- | --- | --- | --- | --- |
|  | Nr. (%) | Nr. (%) |  | Nr. (%) | Nr. (%) |
| KPS <80% | 2 (18.2%) | 9 (81.8%) | KPS <90% | 25 (69.4%) | 11 (30.6%) |
| KPS ≥80% | 1 (1.3%) | 76 (98.7%) | KPS ≥90% | 25 (48.1%) | 27 (51.9%) |
|  |  |  | Trastuzumab BC therapy | 19 (73.1%) | 7 (26.9%) |
|  | **Dural affection** | **No dural affection** | No Trastuzumab BC therapy | 31 (50.0%) | 31 (50.0%) |
| Age at BC diagnosis ≥65years | 5 (31.3%) | 12 (68.7%) | TI BC-BM <3 years | 29 (72.5%) | 11 (27.5%) |
| Age at BC diagnosis <65years | 42 (58.3%) | 29 (41.7%) | TI BC-BM ≥3 years | 21 (43.8%) | 27 (56.2%) |
| Trastuzumab BC therapy | 19 (76.0%) | 6 (24.0%) | BM ER RS negative | 29 (67.4%) | 14 (32.6%) |
| No Trastuzumab BC therapy | 28 (44.4%) | 35 (55.6%) | BM ER RS positive | 21 (46.7%) | 24 (53.3%) |
| Infratentorial BM | 23 (69.7%) | 10 (30.3%) | Preop. WBC (>10/nl) | 28 (75.7%) | 9 (24.3%) |
| Supratentorial BM | 24 (43.6%) | 31 (56.4%) | Preop. WBC (≤10/nl) | 20 (44.4%) | 25 (55.6%) |
| BM HER2 RS positive | 25 (69.4%) | 11 (30.6%) |  |  |  |
| BM HER2 RS negative | 22 (42.3%) | 30 (57.7%) |  | **Edema >10mm** | **Edema ≤10mm** |
|  |  |  |  |  |  |
|  | **Circular CE** | **No circular CE** | Extracranial metastases | 24 (68.6%) | 11 (31.4%) |
| Age at BC diagnosis ≥65years | 9 (56.3%) | 8 (43.7%) | No extracranial metastases | 46 (88.5%) | 6 (11.5%) |
| Age at BC diagnosis <65years | 10 (13.9%) | 61 (86.1%) | TI BC-BM <5 years | 48 (88.9%) | 6 (11.1%) |
| Trastuzumab BC therapy | 1 (4.0%) | 24 (96.0%) | TI BC-BM ≥5 years | 22 (66.7%) | 11 (33.3%) |
| No Trastuzumab BC therapy | 18 (28.6%) | 45 (71.4%) | BM ER RS negative | 39 (90.7%) | 4 (9.3%) |
| BC HER2 RS negative | 13 (28.3%) | 33 (71.7%) | BM ER RS positive | 32 (71.1%) | 13 (28.9%) |
| BC HER2 RS positive | 3 (9.4%) | 29 (90.6%) | HR RC identic | 35 (89.7%) | 4 (10.3%) |
| BM HER2 RS negative | 16 (30.8%) | 36 (69.2%) | HR RC converted | 27 (69.2%) | 12 (30.8%) |
| BM HER2 RS positive | 3 (8.3%) | 33 (91.7%) |  |  |  |
| BM ER RS negative | 16 (37.2%) | 27 (62.8%) |  |  |  |
| BM ER RS positive | 3 (6.7%) | 42 (93.3%) |  |  |  |
|  |  |  |  |  |  |
|  | **Cystic components** | **No cystic components** |  |  |  |
| BM ER RS negative | 18 (41.9%) | 25 (58.1%) |  |  |  |
| BM ER RS positive | 5 (11.1%) | 40 (88.9%) |  |  |  |

Abbreviations: Nr.: number, BM: brain metastases, KPS: Karnofsky Performance Status scale, BC: breast cancer, CE: contrast-enhancement, HER2: human epidermal growth factor receptor 2, TI: time interval, ER: estrogen receptor, HR: hormone receptors (=ER and PR), RS: receptor status, RC: receptor conversion, preop.: preoperative, WBC: white blood cells

**Supplementary table 3:** Univariate cox regression analysis of OS

| **Parameter** | **p-value** | **HR** | **95% CI** |
| --- | --- | --- | --- |
| **Clinical parameters** | | | |
| Age at BC diagnosis ≥65 years | 0.065 | 1.82 | 0.96-3.43 |
| Age at BM diagnosis ≥65 years | 0.171 | 1.41 | 0.86-2.30 |
| TI BC-BM < 3years | 0.623 | 1.13 | 0.69-1.86 |
| KPS <80 | **0.001** | 3.70 | 1.74-7.86 |
| Surgical treatment of BC (Mastectomy) | 0.488 | 1.19 | 0.73-1.93 |
| Trastuzumab as BC therapy | 0.083 | 0.60 | 0.34-1.07 |
| Extracranial metastases | 0.710 | 1.10 | 0.68-1.78 |
| BM location | 0.094 | 1.52 | 0.93-2.48 |
| Number of BM | 0.159 | 1.43 | 0.87-2.36 |
| Preop. Seizure | 0.069 | 6.66 | 0.87-51.21 |
| Postop. Seizure | 0.457 | 0.78 | 0.41-1.50 |
| Arterial hypertonia | 0.389 | 1.18 | 0.72-1.92 |
| DM | 0.485 | 0.66 | 0.21-2.12 |
| Preop. WBC>10/nl | 0.086 | 1.57 | 0.94-2.61 |
| Preop. LDH >247U/l | 0.067 | 1.71 | 0.96-3.02 |
| **Immunohistochemically parameters** | | | |
| BM HER2 RS negative | **0.017** | 1.90 | 1.12-3.21 |
| BM ER RS negative | 0.369 | 1.25 | 0.77-2.03 |
| BM PR RS negative | 0.453 | 0.80 | 0.46-1.42 |
| BC HER2 RS negative | 0.092 | 1.62 | 0.92-2.85 |
| HER2 RC converted | 0.389 | 1.42 | 0.64-3.16 |
| HR RC identic | 0.149 | 1.47 | 0.87-2.49 |
| **Radiological parameters** | | | |
| Hemorrhage | 0.795 | 1.17 | 0.36-3.84 |
| Diameter of BM >30mm | 0.294 | 1.30 | 0.80-2.10 |
| Dural affection | 0.492 | 0.84 | 0.52-1.37 |
| Circular CE | 0.844 | 1.06 | 0.59-1.92 |
| Cystic components | 0.290 | 0.74 | 0.43-1.30 |
| Necrosis | **0.051** | 1.66 | 1.00-2.76 |
| Edema >10mm | 0.597 | 1.18 | 0.64-2.18 |
| Midline shift | 0.587 | 1.18 | 0.64-2.18 |
| Intraventricular lesion | 0.740 | 1.40 | 0.19-10.27 |
| Ventricular infiltration | 0.412 | 1.40 | 0.63-3.09 |
| Ventricular contact | 0.311 | 1.35 | 0.76-2.41 |

Abbreviations: HR: hazard ratio, TI: time interval, BC: breast cancer, BM: brain metastasis, OS: overall survival, RS: receptor status, HER2: human epidermal growth factor receptor 2, ER: estrogen receptor, PR: progesterone receptor, HR: hormone receptors (=ER and PR), RC: receptor conversion, CE: contrast-enhancement, WBC: white blood cells, LDH: lactat dehydrogenase DM: diabetes mellitus, preop.: preoperative, postop.: postoperative
